# Blood-Based Proteomic Profiling Identifies Potential Biomarker Candidates and Pathogenic Pathways in Dementia

**DOI:** 10.3390/ijms24098117

**Published:** 2023-05-01

**Authors:** Hanan Ehtewish, Areej Mesleh, Georgios Ponirakis, Alberto De la Fuente, Aijaz Parray, Ilham Bensmail, Houari Abdesselem, Marwan Ramadan, Shafi Khan, Mani Chandran, Raheem Ayadathil, Ahmed Elsotouhy, Ahmed Own, Hanadi Al Hamad, Essam M. Abdelalim, Julie Decock, Nehad M. Alajez, Omar Albagha, Paul J. Thornalley, Abdelilah Arredouani, Rayaz A. Malik, Omar M. A. El-Agnaf

**Affiliations:** 1 College of Health and Life Sciences (CHLS), Hamad Bin Khalifa University (HBKU), Qatar Foundation (QF), Doha P.O. Box 34110, Qatar; 2 Neurological Disorders Research Center, Qatar Biomedical Research Institute (QBRI), Hamad Bin Khalifa University (HBKU), Qatar Foundation (QF), Doha P.O. Box 34110, Qatar; 3 Department of Medicine, Weill Cornell Medicine-Qatar, Qatar Foundation (QF), Doha P.O. Box 24144, Qatar; 4 Diabetes Research Center, Qatar Biomedical Research Institute (QBRI), Hamad Bin Khalifa University (HBKU), Qatar Foundation (QF), Doha P.O. Box 34110, Qatar; 5 The Neuroscience Institute, Academic Health System, Hamad Medical Corporation (HMC), Doha P.O. Box 3050, Qatar; 6 Proteomics Core Facility, Qatar Biomedical Research Institute (QBRI), Hamad Bin Khalifa University (HBKU), Qatar Foundation (QF), Doha P.O. Box 34110, Qatar; 7 Geriatric and Memory Clinic, Rumailah Hospital, Hamad Medical Corporation (HMC), Doha P.O. Box 3050, Qatar; 8 Department of Clinical Radiology, Weill Cornell Medicine-Qatar, Qatar Foundation, Doha P.O. Box 24144, Qatar; 9 Neuroradiology Department, Hamad General Hospital, Hamad Medical Corporation, Doha P.O. Box 3050, Qatar; 10 Translational Cancer and Immunity Center, Qatar Biomedical Research Institute (QBRI), Hamad Bin Khalifa University (HBKU), Qatar Foundation (QF), Doha P.O. Box 34110, Qatar

**Keywords:** dementia, MCI, plasma proteomics, biomarkers, Olink assay, machine learning

## Abstract

Dementia is a progressive and debilitating neurological disease that affects millions of people worldwide. Identifying the minimally invasive biomarkers associated with dementia that could provide insights into the disease pathogenesis, improve early diagnosis, and facilitate the development of effective treatments is pressing. Proteomic studies have emerged as a promising approach for identifying the protein biomarkers associated with dementia. This pilot study aimed to investigate the plasma proteome profile and identify a panel of various protein biomarkers for dementia. We used a high-throughput proximity extension immunoassay to quantify 1090 proteins in 122 participants (22 with dementia, 64 with mild cognitive impairment (MCI), and 36 controls with normal cognitive function). Limma-based differential expression analysis reported the dysregulation of 61 proteins in the plasma of those with dementia compared with controls, and machine learning algorithms identified 17 stable diagnostic biomarkers that differentiated individuals with AUC = 0.98 ± 0.02. There was also the dysregulation of 153 plasma proteins in individuals with dementia compared with those with MCI, and machine learning algorithms identified 8 biomarkers that classified dementia from MCI with an AUC of 0.87 ± 0.07. Moreover, multiple proteins selected in both diagnostic panels such as NEFL, IL17D, WNT9A, and PGF were negatively correlated with cognitive performance, with a correlation coefficient (r^2^) ≤ −0.47. Gene Ontology (GO) and pathway analysis of dementia-associated proteins implicated immune response, vascular injury, and extracellular matrix organization pathways in dementia pathogenesis. In conclusion, the combination of high-throughput proteomics and machine learning enabled us to identify a blood-based protein signature capable of potentially differentiating dementia from MCI and cognitively normal controls. Further research is required to validate these biomarkers and investigate the potential underlying mechanisms for the development of dementia.

## 1. Introduction

Dementia is a neurodegenerative disease initiated many years before the appearance of clinical deficits of memory, thinking and reasoning, and behavioral changes. Alzheimer’s disease (AD) is the most common form of dementia, affecting 10% of the population aged ≥ 65 years and contributing to 60–80% of dementia cases [1,2,3]. The World Alzheimer Report 2015 estimated that 46.8 million people worldwide were living with dementia in 2015, which is estimated to reach 74.7 million by 2030 [4]. The rising life expectancy contributes to an increase in the aged population and, consequently, an increase in individuals with cognitive dysfunction [5], resulting in an enormous burden on caregivers, communities, and health and social care services [6]. AD has a complex, multifactorial pathology that follows a continuum of conditions, ranging from long preclinical asymptomatic to mild cognitive impairment (MCI) to dementia [7,8]. MCI is a heterogeneous condition, described as an intermediate stage between the expected decline in cognitive function due to normal aging and dementia but with minimal impact on everyday activities [9]. Typically, MCI affects memory, language, thinking, and judgment ability. The prevalence of MCI increases with age; it is estimated that 7% of individuals aged 60–64 years are affected, while approximately 25% of people aged 80–84 may develop cognitive impairment [10]. About 50% of the MCI population are at risk of developing dementia, while others either remain as MCI or return to normal cognition status [11].

The clinical methods currently used for the detection of dementia require multiple-domain neuropsychological testing, CT- or MRI-based evaluation of brain atrophy, positron emission tomography (PET) for amyloid deposits, and CSF biomarker analysis [12,13]. However, these methods are invasive, costly, and/or limited for community-based population screening, as they require on-site technologies and expertise in specialized centers. The recent discovery of classic AD pathology blood biomarkers, namely plasma Aβ42/40 ratios, phosphorylated tau (p-tau), and the neurodegeneration biomarker neurofilament light polypeptide (NEFL), raises the possibility of establishing alternative, highly sensitive/specific, and less invasive blood-based diagnostic panels of proteins reflecting the pathological pathways underlying the development of dementia [14,15,16,17,18,19,20,21]. In particular, plasma NEFL has been extensively reported to be associated with the risk of developing different types of dementia, including AD [22], worse global cognition and memory and brain imaging changes [23], and disease progression [24,25]. Recent advances in ultrasensitive and high-throughput proteomic technologies such as Olink proximity extension immunoassay (PEA) are promising approaches in the search for comprehensive protein profiling and novel biomarker discovery [26,27]. As some proteomic studies have revealed the dysregulation of AD-associated biomarkers in plasma using PEA, only a few protein panels have been screened [25,28,29,30], and studies using the same platform have not been conducted in Middle Eastern populations. Comprehensive protein profiling is needed to validate and clarify the protein signatures of dementia in the blood and delineate the pathogenetic pathways that are not just limited to the Aβ cascade and tauopathy but also reflect other processes that are directly and/or indirectly involved in the pathophysiology of dementia such as cerebrovascular changes, neuroinflammation, mitochondrial dysfunction, and oxidative stress [31].

**Figure 1:**
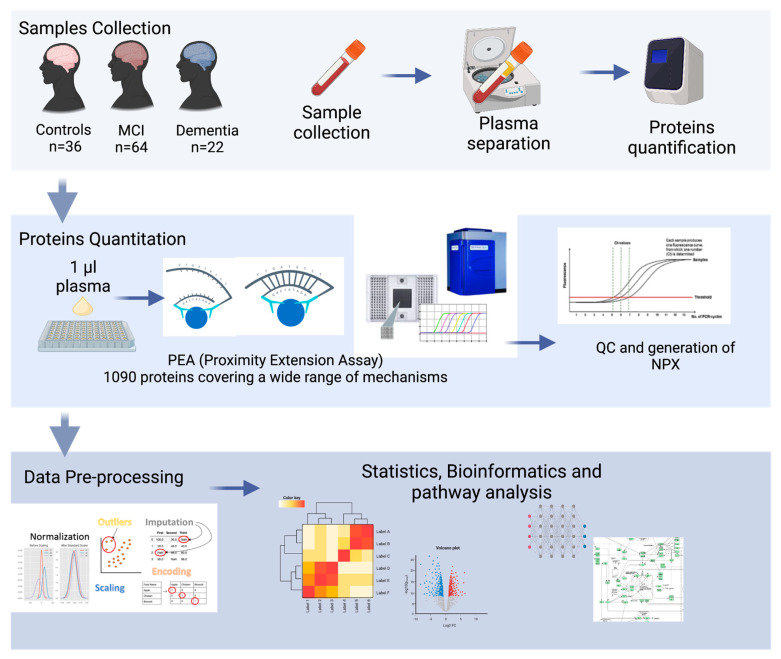
Overview of this study’s experimental design: plasma from 22 dementia patients, 64 MCI patients, and 36 healthy subjects were isolated. proteomic analysis (PAE) was elaborated with bioinformatics to identify the potential biomarkers and their associated pathways.

In this pilot study, the highly sensitive PEA platform was applied to systematically evaluate the plasma protein profiles of 122 individuals, quantifying 1090 plasma proteins. The main aim of this study was to explore the proteomic profile of a Middle Eastern population with MCI, dementia, and normal cognitive function and to identify a combination of plasma biomarkers that could accurately classify dementia from MCI and cognitively normal controls and characterize the underlying pathological pathways of dementia. An overview of this study’s design is shown in Figure 1.

## 2. Results

### 2.1. Characteristics of the Study Participants

The study population included a cross-sectional cohort of 122 participants with dementia, MCI, and normal cognitive function recruited from the Geriatric and Memory clinic in Rumailah Hospital (Doha, Qatar), between 2019 and 2021. The subjects with dementia and MCI fulfilled the International Classification of Diseases, Tenth Revision (ICD-10) criteria. Of the 122 subjects, 22 patients had dementia, 64 had MCI, and 36 were cognitively normal controls (Table 1). The participants were 75.8 ± 4.97, 69.8 ± 8.06, and 67.2 ± 7.34 years old, respectively. Approximately 50% of MCI and control subjects were female, but the majority of dementia cases were male. Subjects with dementia had an average MoCA score of 11.2 ± 6.53, and 23.8% had a college education or more. In comparison, subjects with MCI had an average MoCA score of 23.0 ± 16.56, and 40.3% had a college education. For the control group, the average MoCA score was 28.9 ± 1.56, with 43.3% having a college education or more. The MoCA scores were significantly different (adjusted *p* < 0.05) between dementia, MCI, and the control participants (dementia vs. control *p*-value = 9.95 × 10^−13^, dementia vs. MCI *p*-value = 2.23 × 10^−4^, and MCI vs. control *p*-value = 2.21 × 10^−6^).

### 2.2. Differentially Expressed Plasma Biomarkers in Dementia

**Figure 2:**
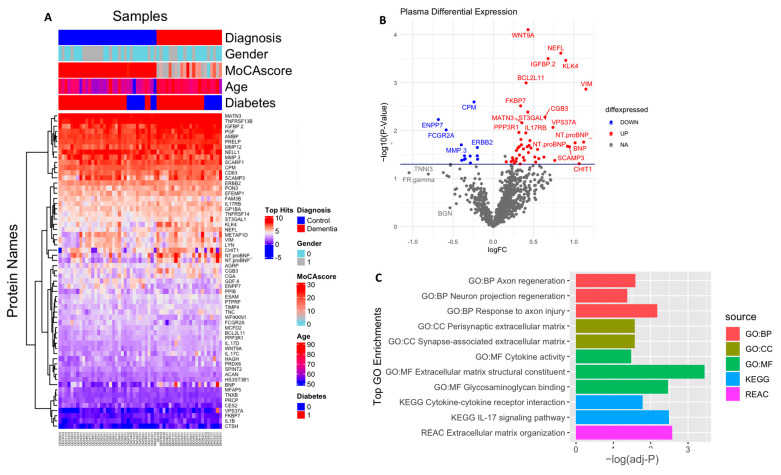
Differential expression of plasma proteome (dementia vs. control): (**A**) Supervised cluster analysis across the control and dementia samples using the 61 significantly altered proteins in the dataset (*p* < 0.05); (**B**) volcano plot displaying the log2 fold change (x-axis) against the limma-derived −log10 statistical *p*-value (y-axis) for all proteins differentially expressed between control and dementia cases of the plasma proteome. Proteins with significantly decreased levels in dementia (*p* < 0.05) are shown in blue, while the proteins with significantly increased levels in disease are noted in red. Select proteins are labeled; (**C**) representative Gene Ontology (GO) terms associated with significantly altered proteins in dementia in the domains of biological process, molecular function, cellular component, and KEGG and REAC pathway are shown.

**Figure 3:**
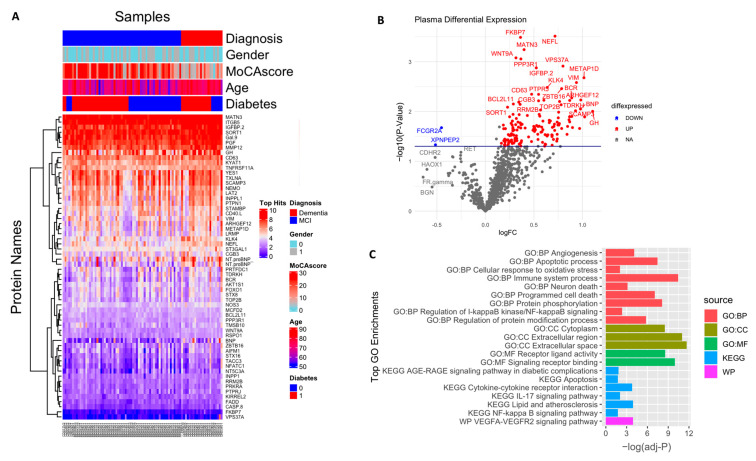
Differential expression of plasma proteome (dementia vs. MCl): (**A**) Supervised cluster analysis across the control and dementia samples using the 152 significantly altered proteins in the dataset (*p* < 0.05); (**B**) volcano plot displaying the log2 fold change (x-axis) against the limma-derived −log10 statistical *p*-value (y-axis) for all proteins differentially expressed between dementia and MCI cases of the plasma proteome. Proteins with significantly decreased levels in dementia (*p* < 0.05) are shown in blue, while the proteins with significantly increased levels in disease are noted in red. Selected proteins are labeled; (**C**) representative Gene Ontology (GO) terms associated with significantly altered proteins in dementia in the domains of biological process, molecular function, cellular component, and KEGG and REAC pathway are shown.

In an exploratory analysis to identify the putative biomarkers associated with dementia, we identified differentially expressed proteins between dementia and cognitively normal controls. Among the 1090 plasma proteins, 60 were significantly altered in subjects with dementia with *p* < 0.05 (Figure 2A,B; Table S1), comprising 47 significantly upregulated and 13 significantly downregulated proteins. Seventeen proteins had more than a 50% difference between subjects with dementia and controls, including VIM (2.2 fold increase), NTproBNP (2.2 fold increase), CHIT1 (2.2 fold increase), NEFL (1.7 fold increase), ENPP7 (1.6 fold decrease), and FCGR2A (1.5 fold decrease). We also identified the difference in the plasma protein expression profile between subjects with MCI and dementia. There were a total of 153 differentially expressed proteins between subjects with MCI and dementia, 149 of which were upregulated with *p* < 0.05 (Figure 3A,B; Table S2). In addition, 68 proteins showed a greater than 1.5-fold change difference between dementia and MCI. Interestingly, 31 proteins overlapped with the biomarkers that differed between subjects with dementia and controls.

### 2.3. Gene Ontology and Pathway Analysis

Gene Ontology (GO) analysis was performed to identify enriched functional categories among the 61 altered dementia-associated plasma proteins (Figure 2C, Table S3). Nine proteins revealed that the pathway related to extracellular matrix (ECM) organization was dysregulated in dementia (adjusted *p*-value = 0.002) based on the KEGG database. Seven of these were enriched in the ECM structural constituent (adjusted *p*-value = 0.0003), namely MATN3, TNC, EFEMP1, ACAN, PRELP, TNXB, and MFAP5. Additionally, PGF, PTPRF, ACAN, PRELP, TNXB, NELL1, and GDF8 were enriched in glycosaminoglycan binding (adjusted *p*-value = 0.003). ACAN and TNC were particularly involved in the presynaptic ECM and synapse-associated ECM (adjusted *p*-value = 0.02, 0.026, respectively). In addition, the molecules associated with cell adhesion that might interact with the ECM were over-represented and included BCL2L11, PTPRF, ERBB2, SCARF1, LYN, ILB1, and SPINT2, which were also linked to inflammation and response to axon injury, axon regeneration, and neuronal regeneration. Moreover, mostly upregulated immune-related proteins such as IL17C, IL17RB, IL1B, IL17D, NFRSF13B, and TNFRSF14 were involved in IL-17 signaling and cytokine–cytokine receptor interaction pathways (adjusted *p*-value = 0.003 and 0.01, respectively), according to the KEGG and Reactome pathway databases, respectively. Additionally, the cell component revealed a high number of proteins present in the extracellular region, ECM, and extracellular exosome; detailed results are included in Table S3.

The enrichment analysis of the 153 dysregulated proteins in subjects with dementia compared with those in individuals with MCI (Figure 3C, Table S4) showed that they are mainly enriched in the immune system (adjusted *p* = 3.54 × 10^−11^); 15 of these proteins were shown to be involved in the cytokine–cytokine receptor interaction pathway (adjusted *p*-value = 0.0001), including CCL28, CCL27, CXCL13, CD40, IL4R, IL1B, IL17C, IL17D, TNFRSF11A, TNFRSF14, and TNFRSF13B. The cytokines IL17D, IL17C, and IL1B, in addition to another four markers, namely CASP8, CASP3, FADD, and IKBKG, were found to be involved in the IL-17 signaling pathway (adjusted *p*-value = 0.009). Moreover, another subset of proteins was associated with the apoptotic process and programmed cell death (adjusted *p*-value = 3.43 × 10^−8^, *p*-value = 8.28 × 10^−8^, respectively), in which 14 predominantly upregulated proteins were involved in neuronal death (adjusted *p*-value = 0.0007), including NEFL, BCL2L11, FADD, CASP3, CASP8, and FPXO3. In addition, some vascular injury-related proteins were enriched in angiogenic pathways (adjusted *p*-value = 8.42 × 10^−5^) and involved in the VEGFA–VEGFR2 signaling pathway (adjusted *p*-value = 0.0001), including PGF, PTPRJ, NOS3, ADM, and NTproBNP. Moreover, the dysregulation of the NF-kappa-B signaling pathway (adjusted *p*-value = 0.01) and the AGE/RAGE pathway (adjusted *p*-value = 0.03) was also shown. Other biological processes were found to be over-represented, including signal transduction, the regulation of protein phosphorylation and modification process, response to stress, cell communication, and cell adhesion. Additionally, the cell component showed the enrichment of several proteins in the extracellular region, cytoplasm, and cytosol. A detailed description is included in Table S4. 

### 2.4. Multivariate Diagnostic Performance of Selected Plasma Protein Biomarkers

**Figure 4:**
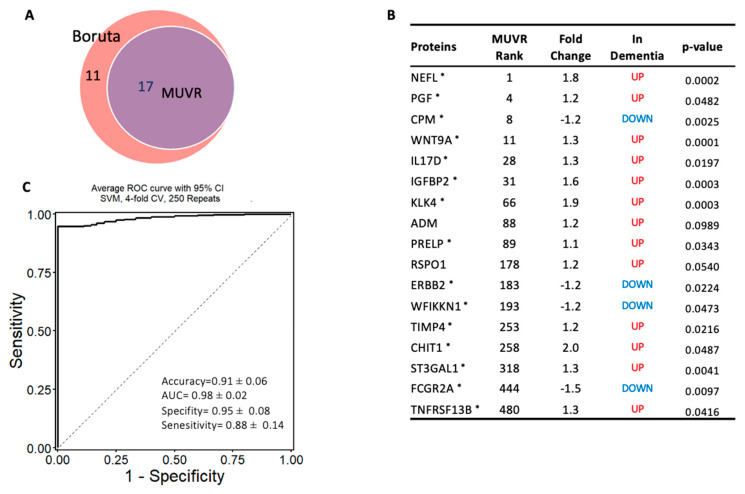
Machine learning model to discriminate dementia from controls: (**A**) Two-variable (feature) selection algorithms were used to select the most robust proteins to differentiate dementia from cognitively normal controls; MUVR (multivariate modeling with minimally biased variable selection) and Boruta (a wrapper algorithm for all relevant feature selection and feature importance with random selection runs); (**B**) the predictive variables selected using MUVR and Boruta (Panel A). A total of 17 plasma proteins, with MUVR rank, fold change, and *p*-value. Red and blue indicate up- and downregulated plasma proteins, respectively; those with * indicate the limma-identified significant proteins (*p*-value < 0.05); (**C**) the ROC curve of the model using the 17 candidates’ variables. SVM outcome shows the trade-off between the true-positive rate (sensitivity) and false-positive rate (1–pecificity) for different classification thresholds.

To identify the most robust markers for the classification of dementia, independently of the differential expressed proteins, a machine learning algorithm MUVR and Boruta were used on the complete proteomic profile of 1090 proteins. MUVR identified a minimal–optimal set of seventeen proteins (Panel A). This set of biomarkers was also selected by Boruta, thereby confirming that they are the most stable markers to classify dementia from cognitively normal subjects (Figure 4A,B). In terms of the classification algorithm, support vector machines (SVMs) were selected, as they demonstrated the strongest predictive performance of the models (Figures S1 and S2). This model of 17 stable proteins had a high potential to be applied as a diagnostic panel for dementia, as it accurately distinguished subjects with dementia from cognitively normal controls with high accuracy of 91% (AUC = 0.98 ± 0.03; specificity = 95%, and sensitivity = 88%; Figure 4C). Notably, 15 out of the 17 multivariate model proteins were differentially expressed in dementia (*p* < 0.05) in limma, such as NEFL, CPM, WNT9A, IL17D, IGFBP2, KLK4, PRELP, and PGF, Figure 4B.

**Figure 5:**
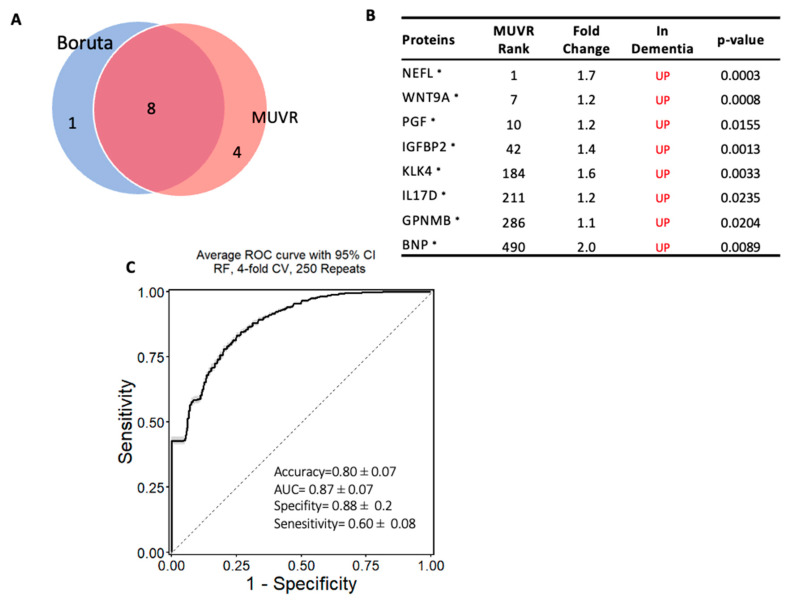
Machine learning model to discriminate dementia from MCI: (**A**) Two-variable (feature) selection algorithms were used to select the most robust proteins to differentiate dementia from MCI cases; MUVR (multivariate modeling with minimally biased variable selection) and Boruta (a wrapper algorithm for all relevant feature selection and feature importance with random selection runs); (**B**) the predictive variables selected using MUVR and Boruta (Panel B). A total of 8 plasma proteins, with MUVR rank, fold change, and *p*-value. Red and blue indicate up- and downregulated plasma proteins, respectively. Those with * indicate the limma-identified significant proteins (*p*-value < 0.05); (**C**) the ROC curve of the model using the 8 candidates’ variables. SVM outcome shows the trade-off between the true-positive rate (sensitivity) and false-positive rate (1–specificity) for different classification thresholds.

The same approach was used to establish another model to classify subjects with dementia from MCI, and a minimal–optimal set of 12 proteins was selected using MUVR, 8 of which had been confirmed using Boruta. This result shows that these eight proteins are the most stable to differentiate dementia from MCI and are considered a potential diagnostic panel (Panel B) (Figure 5A,B). These eight proteins were also significantly altered in dementia compared with MCI based on the limma analysis. The eight-protein model moderately differentiated those with dementia from those with MCI, with an accuracy of 80% (AUC = 0.87 ± 0.07; specificity = 88% and sensitivity = 60%; Figure 5C). Notably, NEFL, WNT9A, IL17D, IGFBP2, KLK4, and PGF proteins were included in both models that differentiated dementia from either cognitively normal controls or MCI.

### 2.5. Multiple Differentially Regulated Plasma Proteins Associated with Cognitive Performance

**Figure 6:**
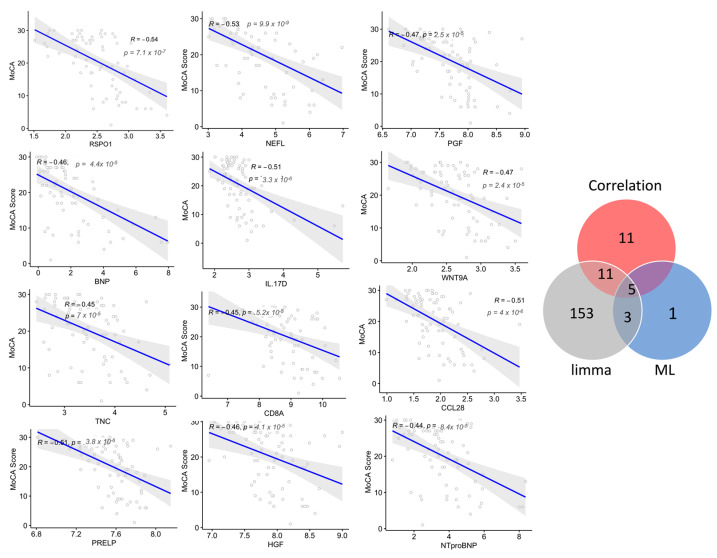
Correlations between the plasma levels of the proteins and cognitive decline are indicated by Montreal Cognitive Assessment (MoCA) scores. The top correlated proteins with Spearman’s correlation coefficients (r^2^) and the associated *p*-values are shown. Venn diagram shows the number of correlated proteins that overlapped with the selected variable of differentially expressed proteins using limma and machine learning (ML) algorithms in dementia compared with MCI.

Next, to examine whether a panel of proteins was associated with disease progression, the correlation of the plasma protein expression level (NPX values) with cognitive performance (MoCA scores) in subjects with dementia and MCI was analyzed. The levels of 27 plasma proteins negatively correlated with the MoCA score, with a correlation coefficient (r^2^) between −0.40 and −0.53 (*p* < 0.05; Figure 6, Table S5); among these proteins, 14 markers were dysregulated in subjects with dementia compared with MCI. Six of these proteins were part of the diagnostic Panel B constructed using MUVR and Boruta, namely NEFL, IL17D, WNT9A, GPF, BNP, and IGFBP2, which had a strong negative correlation, with r^2^ ≤ −0.45. The correlation analysis showed only three proteins with a moderate positive correlation with MoCA scores, with r^2^ of 0.3. Interestingly, the further examination of how these plasma proteins were dysregulated in relation to cognitive decline showed that the top six proteins correlated with the MoCA score (r^2^ < −0.45, *p*-value < 0.05), namely RSPO1, NEFL, IL17D, CCL28, WNT9A, and PGF, and they were dysregulated in those with more advanced dementia with severe cognitive deficits (MoCA score < 15). Notably, NEFL was the only protein altered in both late and intermediate stages (MoCA score 16–25) but not in the mild stage of cognitive decline (MoCA score > 25). Interestingly, this panel of proteins, except RSPO1 and CCL28, were also identified using the machine learning multivariate model (diagnostic Panel B) that discriminated dementia from MCI, with an AUC = 0.87, (Figure 5). GO was conducted on the proteins with r^2^ of ≤ −0.4 and a significant *p*-value. Notably, in agreement with the GO enrichment analysis of the differentially expressed proteins in dementia compared with MCI, the set of correlated proteins was significantly enriched in the cytokine–cytokine receptor interaction pathway based on the KEGG database. Additionally, several biological processes were over-represented, including the regulation of IL6 and IL8 production, apoptosis, angiogenesis, protein phosphorylation, and cellular response to stress (Table S6).

## 3. Discussion

Identifying a blood-based biomarker for dementia is urgently needed for disease risk prediction and diagnosis. These biomarkers not only expand our understanding of the underlying pathophysiology of dementia but also drive us toward effective therapeutic strategies. Thus, we applied a high-throughput immunoassay technique to explore the plasma proteome profile of 1090 proteins in a cohort of people with dementia. Certainly, the PEA method is more reliable, reproducible, and convenient; however, it is limited in terms of the number of proteins that can be detected, compared with that of the human proteome. In this study, in addition to the well-known marker of neurodegeneration NEFL, our findings highlight a set of promising candidate biomarkers that require further validation in independent disease cohorts. Furthermore, machine learning algorithms identified a large number of altered biomarkers that highly accurately identified those with dementia. The subsequent correlation of the plasma proteins with the severity of cognitive dysfunction revealed a group of potential biomarkers for disease progression. Our enrichment and pathway analyses revealed biomarkers in several pathways that might be associated with the pathophysiology of dementia, including immune response, vascular injury, and extracellular matrix organization. Our extensive plasma biomarker screening identified 61 altered proteins in the blood of subjects with dementia compared with controls. Seventeen proteins were identified as potential diagnostic biomarkers (Panel A) for dementia, as they highly accurately distinguished those with dementia from cognitively normal controls, with high accuracy, sensitivity, and specificity. Notably, a comparison of our findings to a previous PEA-based proteomic study that assayed 1060 proteins in a Hong Kong Chinese population with AD revealed 28 overlapped proteins; of these LYN, KLK4, VPS37A, and NELL1 were also identified as hub proteins representing an AD plasma proteomic profile [32]. Interestingly, in our study, KLK4 and LYN were identified as among the best discriminative plasma proteins for dementia in the MUVR model. NEFL and TIMP4 were also consistently dysregulated in all types of dementia in a PEA-based study in the Massachusetts Alzheimer’s Disease Research Center cohort [25]. Consistent with our finding, a study assayed 270 proteins in the CSF and plasma of Swedish subjects (BioFINDER cohort) using the PEA platform and reported alterations in plasma levels of HAGH and TIMP4, and the CSF levels of CHIT1, TNFRSF14 in AD patients [29]. Additionally, in the same study, 3 proteins in our diagnostic panel (IGFBP2, TIMP4, and RSPO1) were also selected in the regression model of 74 plasma proteins that classified AD dementia, and another 3 proteins identified using our classification model (RSPO1, CHIT1, and WFIKKN1) were also selected in 36 CSF biomarker panels that accurately identified AD dementia [29]. We identified a high expression level of AGRP and NTproBNP in the plasma of patients with dementia; a SOMAmer-based study also reported the association of these proteins with a high risk of dementia [33]. Moreover, consistent with our findings, a previous study revealed that plasma BNP was significantly elevated in patients with AD; additionally, it showed that BNP was one of the five markers that differentiated those with AD dementia and correlated with the CSF concentration of β-amyloid 1–42 and p-tau [34]. We also identified eight proteins as potential biomarkers (Panel B) that could distinguish those with dementia from MCI with high accuracy and specificity. Five proteins out of the eight proteins using the multivariate model were differentially expressed in dementia (*p* < 0.05) and negatively correlated with cognitive performance scores (r^2^ between −0.53 and −0.46), namely NEFL, WNT9A, IL17D, PRELP, and PGF. Consistent with our findings, XIN1, PECAM1, CD40, and JAMA were also reported to be differentially expressed in the plasma of subjects with dementia compared with MCI [29]. Interestingly, six proteins (NEFL, WNT9A, IL17D, IGFBP2, KLK4, and PGF) were repeatedly selected in both diagnostic models that classified dementia from both cognitively normal controls and MCI.

This study highlights a set of candidate biomarkers of neurodegeneration and other pathways that seem to be involved in the pathogenesis of dementia and therefore deserve further validation. Our findings highlight the upregulation of several functionally immune-related proteins in dementia, such as IL17D, IL17C, IL17RB, and IL1B, among which IL17D also showed a strong negative correlation with cognitive performance. IL-17D is a pro-inflammatory cytokine that belongs to the IL-17 family, plays a role in the immune response, and enhances the production of other pro-inflammatory cytokines, such as TNF-alpha and IL-1B [35]. Studies have found that the transcriptome levels of two members of the IL17 family (IL17D and IL17RB) were upregulated in human AD astrocytes, suggesting a protective role of members of the IL17 family in AD through the antioxidant-protective Nrf2/IL17D axis against stress [36]. ILD17 is a candidate mediator for ameliorating memory performance trajectory and β-amyloid-induced neuroinflammation in AD [37,38].

That these changes taking place in the dementia brain are detectable in the plasma may reflect the disruption of the blood–brain barrier. Indeed, twenty of the differentially expressed proteins in dementia compared with MCI were enriched in the pathways regulating angiogenesis and involved in the VEGFA–VEGFR2 signaling pathway, including the placental growth factor (PGF). PGF is a member of the vascular endothelial growth factor family, initially identified in the placenta but widely expressed in ischemic or damaged tissues [39], to regulate and repair damaged blood vessels, in addition to having anti-inflammatory and antiapoptotic effects [39,40]. We found increased plasma levels of PGF in those with dementia, and this strongly correlated with cognitive performance scores. The CSF expression level of PGF was also reported to be increased in different types of dementia, including AD and vascular dementia; however, PGF showed higher accuracy in distinguishing frontotemporal dementia [41]. In the context of AD, elevated plasma and cerebral PGF gene expression was associated with worse cognitive trajectories and AD pathology, including the burden of β-amyloid and tau [42,43]. Moreover, signs of cerebrovascular injury, such as white matter lesions, have been associated with higher levels of plasma and CSF PGF in the prodromal stages of AD [44,45].

In our study, two proteins involved in the Wnt signaling pathway (WNT9A and RSPO1) also showed evidence of differential regulation in dementia. The Wnt signaling family plays a role in axonal pathfinding, dendritogenesis, synapse formation, and synaptic plasticity and maintenance [46]. We found a high expression of WNT9A in those with dementia compared with those with MCI and cognitively normal individuals and a strongly negative correlation with the cognitive performance score. In the context of AD, circulating WNT9A was elevated in patients with AD [32], and the WNT9A gene was upregulated in the hippocampi of memory-impaired mice [47]. Indeed, the Wnt signaling pathway, one of the parts of which is WNT9A, has been shown to play a role in AD pathogenesis through the regulation of inflammation, neurogenesis, β-amyloid production, and tau phosphorylation [46,48]. R-spondin 1 (RSPO1) is an activator of the Wnt/β-catenin signaling pathway [49,50]. Our findings showed that plasma levels of RSPO1 were elevated in subjects with dementia and negatively correlated with the MoCA score. It was also one of the predictors in the diagnostic model of dementia. RSPO1 has been identified in regression models to have a high predictive accuracy in both the CSF and blood for AD dementia [29]. It has been reported that presymptomatic and familial AD carriers of mutations in PSEN1 and APP genes have elevated CSF levels of this protein [51]. In recent studies, RSPO1 has also been shown to correlate with hippocampal volume and white matter hyperintensities, indicating that RSPO1 may represent an early pathology marker of neurodegeneration [52]. IGFBP2 has been reported as a potential biomarker, particularly for AD, and we found that IGFBP2 expression levels were higher in participants with dementia than in those with MCI or cognitively normal participants, and it had a moderately negative association with cognitive performance scores. IGFBP-2 is the most abundant insulin-like growth factor (IGF)-binding protein in the brain and is known to regulate the activity of IGFs in this tissue [53]. The elevated levels of IGF-binding proteins, particularly IGFBP-2, impair IGF signaling, thereby inhibiting the neuroprotective effects of IGFs against neurotoxins, including β-amyloid peptide [54,55]. CSF and plasma IGFBP-2 levels are elevated in AD, and plasma levels of IGFBP-2 are associated with smaller hippocampal volumes and brain atrophy [55,56,57,58,59]. Moreover, high plasma levels of IGFBP2 have been shown to predict the risk of MCI conversion to dementia [60,61,62,63].

Certain limitations in our study should be acknowledged, such as the small sample size and age differences in our cohort, limiting the generalizability of our results. This necessitates the confirmation of the identified markers across larger, diverse, well-matched, and independent cohorts. Another limitation is the inability to accurately classify dementia and MCI according to their etiology; thus, future studies incorporating cerebrospinal fluid and PET imaging will be necessary to classify specific neurodegenerative conditions.

## 4. Materials and Methods

### 4.1. Participants and Plasma Collection

This study included plasma samples of a discovery set of 122 participants, comprising 36 cognitively normal controls; 64 subjects with MCI; and 22 subjects with dementia including those with Alzheimer’s dementia (*n* = 3), vascular dementia (*n* = 5), and mixed dementia (*n* = 14). The cohort was collected over three years (2019–2021) and recruited as part of a prospective and longitudinal study in the Geriatric and Memory clinic in Rumailah Hospital (Doha, Qatar). Plasma samples were collected and stored with standardized procedures at the baseline visit. Specifically, blood samples were collected in EDTA tubes and centrifuged at 1500× *g* for 15 min at 4 °C. The plasma was then isolated, collected, and stored at −80 °C. This study was approved by the Qatar Biomedical Research Institute (2019-013), Hamad Medical Corporation (RP14494/14), and Weill Cornell Medicine-Qatar (15-00019), Doha, Qatar, in accordance with the applicable guidelines and regulations. Written, informed consent was collected from all participants.

### 4.2. Diagnostic Procedures

All subjects underwent standardized investigation at a memory disorder unit by geriatricians, geriatric psychiatrists, and neurologists, including clinical assessments and history of illness, caregiver interviews, clinical neurological examination, neuropsychological evaluation, functional history of essential daily living activities, neuroimaging, and laboratory assessments for ruling out other causes of cognitive dysfunction. The diagnosis of MCI and dementia was based on the International Classification of Diseases, Tenth Revision (ICD-10) criteria. Furthermore, the AD diagnosis was based on the typical features of MRI in AD; neuroradiologists assessed the volume loss of the hippocampi, the entorhinal cortex, and the amygdala based on the criteria of Dubois et al. [64] and were blinded to the diagnosis and clinical data. The diagnosis of vascular dementia (VaD) was based on the National Institute of Neurological Disorders and Stroke-Association Internationale pour la Recherche et l’Enseignement en Neurosciences (NINDS-AIREN) criteria [65], which includes multiple large vessel infarcts or single strategically placed infarct (angular gyrus, thalamus, basal forebrain, or posterior (PCA) or anterior cerebral artery (ACA) territories), or multiple lacunes in the basal ganglia and white matter, or extensive periventricular white matter lesions, or combinations thereof. The mixed dementia diagnosis was based on the presence of AD features and significant vascular changes.

### 4.3. Cognitive Function Assessment

Cognitive function was assessed using the Montreal Cognitive Assessment (MoCA) test. The MoCA assesses seven cognitive domains, namely visuospatial/executive, naming, memory, attention, language, abstraction, and delayed recall, giving a total score of 30. A score of ≤26 indicates cognitive impairment. A point was added for individuals who had formal education ≤6th grade. Cognitive symptom duration was estimated from the clinical history obtained from relatives and participants.

### 4.4. Protein Quantification

For the plasma protein quantification, 122 samples were stratified according to the diagnosis. Next, they were allocated, using the randomization generator of Microsoft Excel, to a position on one of two 96-well plates, with 15 uL of each participant’s plasma aliquoted into each well. Protein levels were measured using the validated, highly sensitive, and specific ProSeek^®^ multiplex immunoassay, developed by Olink Proteomics (Uppsala, Sweden) [26,27], at the QBRI core facilities laboratory in Doha, according to the manufacturer’s instructions. Samples were screened using all the human protein targets available in commercial ProSeek^®^ Multiplex 96 target panels at the time of this study (1196 proteins in total), including 6 disease-area-focus panels (cardiovascular II, cardiovascular III, oncology II, oncology III, neurology, and inflammation), complemented by 7 panels focused on important biological processes: cardiometabolic, cell regulation, development, immune response, metabolism, neuro exploratory, and organ damage. In brief, the PEA is a dual-recognition immunoassay, where the plasma antigens bind to matched pairs of oligonucleotide-labeled antibodies. This brings the two antibody probes into close proximity, generating a template for hybridization and extension and enabling amplification with polymerase chain reaction (PCR). The resulting DNA amplicon was then quantified using microfluidic qPCR on a Fluidigm^®^ Biomark instrument. Quality control and normalization were conducted using an internal extension and inter-plate controls, and three samples were disqualified. Moreover, one sample was identified as an outlier due to having a covariate-adjusted PCA value of more than four standard deviations from the centroid (Figure S4). Thus, of the initial 122 participants, 118 were eligible for the analysis. Notably, 105 proteins were excluded due to low detection rates of >30% in cases and controls. Thus, our final statistical analyses included 1090 proteins passing stringent Olink QC measures and detected in >70% of the samples. The missing data for the plasma proteins were imputed using the missForest package in R.

### 4.5. Statistical Analysis

All statistical analysis was performed using R version 4.2.2 (31 October 2022). Continuous clinical variables were reported as the mean ± standard deviation. Differential expression analysis between the groups (dementia versus control and dementia versus MCI) was conducted using the limma (linear models for microarray data) package in R, adjusting for age, gender, and diabetes status. The *p*-values of the multiple comparisons were adjusted using the Benjamini–Hochberg method and the significance threshold of a *p*-value < 0.05. The correlation between the cognitive function scores (MoCA scores) of the participants with dementia and MCI and the plasma biomarkers levels was computed with Spearman’s correlation coefficients (r) using the cor.test() function in the R. All plots were generated using R; the heatmap() function from the R ggplot package was used to generate a heatmap of the top dysregulated plasma proteins, and a volcano plot was generated using the ggplot() function. In addition, the plot.roc() function from the R pROC package was used to generate the ROC curves for the classification models, and the correlation plots ggscatter() function in R was utilized.

### 4.6. Machine Learning Model and ROC Curve Analysis

The minimally unbiased variable selection algorithm (MUVR), a supervised machine learning technique [66], was used to identify the minimal set of variables required for successfully classifying participants of dementia using the complete proteomic profile of the 1090 protein biomarkers. The MUVR algorithm employed a random forest, along with 21 outer and 28 inner cross-validation segments, and a variable ratio of 0.75, to obtain a subset of the minimal–optimal set of molecular features with the strongest biomarker candidates selected (Figures S2A and S3A). In addition, we used the method of Boruta [67], another feature selection method based on RF. Using statistical testing, it iteratively removes the features that are less relevant than randomized features. Boruta was also applied to the 1090 protein biomarkers; the algorithm was run with maxRun = 1000, and all other parameters were set to their default values. To ensure the robustness of the selected variable set, further validation was conducted using several different algorithms within the mlr3 (machine learning in R, version 3) framework [68]. A variety of classification algorithms, including random forests, support vector machines (SVMs), and generalized linear models (GLMs), were applied to our dataset using mlr3, with a four-fold cross-validation procedure of 250 repeats (Figures S2B and S3B). The performance of the models was assessed in terms of accuracy, the area under the receiver operating characteristic curve (AUC), sensitivity, and specificity. Accordingly, the most effective approach for our dataset was selected (Figures S2C and S3C).

### 4.7. Proteins Functional Enrichment Analysis

The enrichment analysis was performed using the g:GOSt function in the online web server g:Profiler to identify the statistically significantly over-represented terms in Gene Ontology (GO), including the cellular component, molecular function, and biological processes. In addition to the GO terminologies, the g:GOSt function includes pathways from KEGG, Reactome, and WikiPathways databases. The significance level for the enriched terms was considered with adjusted *p*-values < 0.05, and the default setting of the g:GOSt function was applied. Differentially expressed proteins with *p*-values < 0.05 for each contrast were included in the enrichment analysis. 

## 5. Conclusions

We systematically studied the plasma proteome of dementia using PEA biomarker technology to quantify 1090 proteins and further employed a machine learning statistical approach to identify the potential plasma-based diagnostic biomarkers. A differential expression analysis revealed 61 and 154 dysregulated plasma proteins in dementia compared with cognitively normal and MCI subjects. Afterward, this study established two highly accurate models of fifteen and eight plasma biomarkers for dementia diagnosis. Moreover, the study sheds light on the underlying mechanistic pathways for dementia, highlighting the role of inflammation, ECM organization, and vascular injury. The significance of these proteomic changes in dementia needs further study.

## Figures and Tables

**Table 1:** Demographic and clinical characteristics of the participants. All data are presented as mean (SD).

	Dementia	MCI	Control
Sample size (*n*)	22	64	36
Gender (F/M)	8/14	29/35	17/19
Mean age in years (SD)	75.8 (4.9)	69.8 (8.0)	67.2 (7.3)
MoCA Score mean (SD)	11.2 (6.5)	23.0 (6.5)	28.9 (1.5)
Duration of cognitive impairment (years)	2.8 (2.0)	2.7 (3.5)	--

MoCA = Montreal Cognitive Assessment.

## Data Availability

All data generated or analyzed during this study are included in this published article (and its Supplementary Information files) or are available from the corresponding author by reasonable request.

## Supplementary Materials

The following supporting information can be downloaded at: https://www.mdpi.com/article/10.3390/ijms24098117/s1.
