# Supplementary material for: Blood-Based Proteomic Profiling Identifies Potential Biomarker Candidates and Pathogenic Pathways in Dementia
Source: Int J Mol Sci. 2023 May 1;24(9):8117. doi: 10.3390/ijms24098117 (PMC10179172; doi:10.3390/ijms24098117)
Supplement: Supplementary file 1 [file ijms-24-08117-s001.zip › Supplementary figures.pdf]

Supplementary figures:

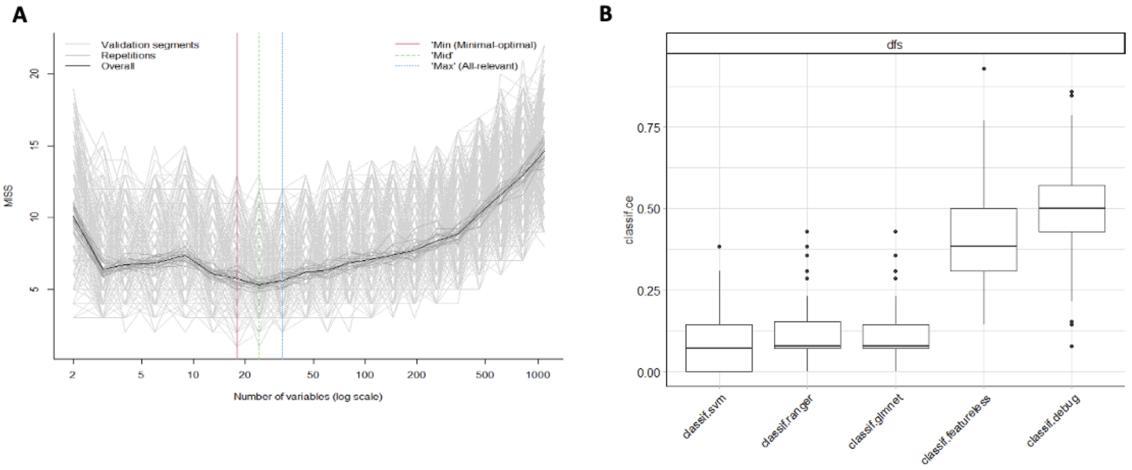

C

learner_id	AUC	Accuracy	TPR	TNR
classif.svm	0.98 ± 0.02	0.91 ± 0.06	0.88 ± 0.14	0.95 ± 0.08
classif.randomforest	0.96 ± 0.05	0.89 ± 0.08	0.92 ± 0.10	0.85 ± 0.15
classif.glmnet	0.96 ± 0.05	0.89 ± 0.07	0.89 ± 0.10	0.89 ± 0.13

Figure S1. Machine learning approach for predictors identification and evaluation (dementia vs controls). A). The misclassification rate as a function of the number of included variables. The light grey lines in the graph represent the validation performance of individual inner segments, while the darker grey lines show the average validation performance of all inner segments. The minimal-optimal ("Min") and all-relevant ("Max") models represent the two extremes of variable selection, with validation performance (measured in misclassifications) minimized within 5% of the minimum. The minimal-optimal model represents the minimum number of variables needed for optimal method performance, such as in biomarker discovery, while the all-relevant model includes all variables with relevant signal-to-noise for the research question, including redundant but non-erroneous variables. The "Mid" model represents a compromise between the "Min" and "Max" models, found at the geometric mean, representing the 17 predictors. **B)** A Comparison of classification error rates for five different algorithms: SVM, RF, GLMnet, Featureless, and Random Guessing on the panel of the 17 proteins identified by MUVR and Boruta. The Featureless algorithm assigns all samples to the largest class, while the Random Guessing algorithm randomly assigns samples to classes. These algorithms are included for comparison. The SVM algorithm showed the best performance, with error rates similar to those of the Random Forests and GLMnet algorithms and much better than Featureless and Random Guessing algorithms. **(C)** A comparison table of performance measures of the five ML methods.

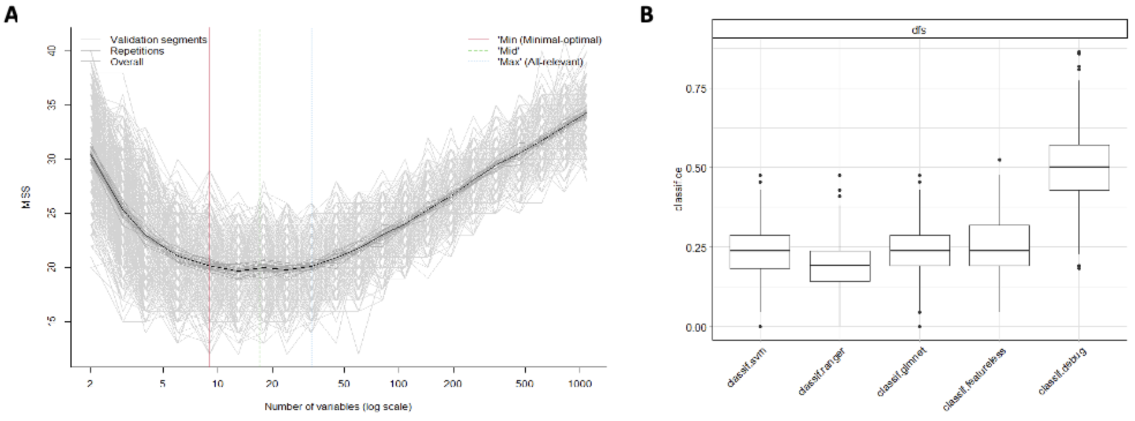

C

learner_id	AUC	Accuracy	TPR	TNR
**classif.svm**	0.84 ± 0.08	0.77 ± 0.08	0.42 ± 0.23	0.90 ± 0.08
**classif.randomforest**	0.87 ± 0.07	0.80 ± 0.07	0.59 ± 0.21	0.88 ± 0.08
**classif.glmnet**	0.83 ± 0.08	0.76 ± 0.08	0.49 ± 0.22	0.86 ± 0.09

Figure S2. Machine learning approach for predictors identification and evaluation (dementia vs MCI). A) The MUVR misclassification rate as a function of the number of included variables. The light grey lines in the graph represent the validation performance of individual inner segments, while the darker grey lines show the average validation performance of all inner segments. The minimal-optimal ("Min") and all-relevant ("Max") models represent the two extremes of variable selection, with validation performance (measured in misclassifications) minimized within 5% of the minimum. The minimal-optimal model represents the minimum number of variables needed for optimal method performance, such as in biomarker discovery, while the all-relevant model includes all variables with relevant signal-to-noise for the research question, including redundant but non-erroneous variables. The "Mid" model represents a compromise between the "Min" and "Max" models, found at the geometric mean, representing the 13 predictors**. B)** A Comparison of classification error rates for five different algorithms: SVM, RF, GLMnet, Featureless, and Random Guessing on the panel of the 8 proteins identified by MUVR and Boruta. The Featureless algorithm assigns all samples to the largest class, while the Random Guessing algorithm randomly assigns samples to classes. These algorithms are included for comparison. The random forest algorithm showed the best performance, with error rates similar to those of the SVM and GLMnet algorithms and much better than Featureless and Random Guessing algorithms. **(C)** A comparison table of performance measures of the five ML methods.

A

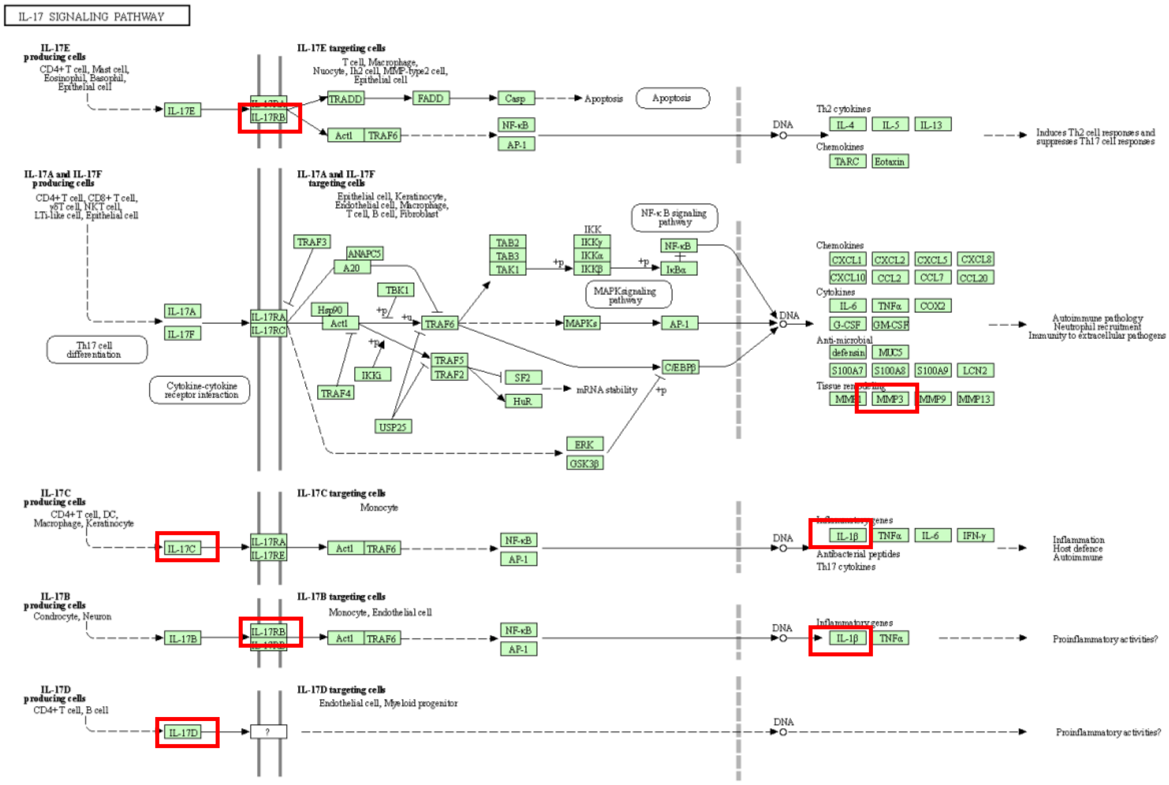
IL-17 SIGNALING PATHWAY
IL-17E producing cells
CD4+ T cell, Mast cell, Eosinophil, Basophil, Epithelial cell
IL-17E
IL-17RB
IL-17E targeting cells
T cell, Macrophage, Nuocyte, Ih2 cell, MMP-type2 cell, Epithelial cell
TRADD
FADD
Casp
Apoptosis
Act1
TRAF6
NF-κB
AP-1
DNA
Th2 cytokines
IL-4
IL-5
IL-13
Chemokines
TARC
Eotaxin
Induces Th2 cell responses and suppresses Th17 cell responses
IL-17A and IL-17F producing cells
CD4+ T cell, CD8+ T cell, γδT cell, NKT cell, LTi-like cell, Epithelial cell
IL-17A
IL-17F
Th17 cell differentiation
Cytokine-cytokine receptor interaction
IL-17A and IL-17F targeting cells
Epithelial cell, Keratinocyte, Endothelial cell, Macrophage, T cell, B cell, Fibroblast
IL-17RA
IL-17RC
TRAF3
ANAPC5
A20
TBK1
Hsp90
TRAF4
IKKi
USP25
TRAF5
TRAF2
SF2
HuR
mRNA stability
TAB2
TAB3
TAK1
IKK
IKKγ
IKKα
IKKβ
NF-κB signaling pathway
IκBα
MAPK signaling pathway
MAPKs
C/EBPβ
ERK
GSK3β
Chemokines
CXCL1
CXCL2
CXCL5
CXCL8
CXCL10
CCL2
CCL7
CCL20
Cytokines
IL-6
TNFα
COX2
G-CSF
GM-CSF
Anti-microbial
defensin
MUC5
S100A7
S100A8
S100A9
LCN2
MMP3
MMP9
MMP13
Autoimmune pathology
Neutrophil recruitment
Immunity to extracellular pathogens
IL-17C producing cells
CD4+ T cell, DC, Macrophage, Keratinocyte
IL-17C
IL-17RA
IL-17RE
IL-17C targeting cells
Monocyte
IL-1β
IFN-γ
Antibacterial peptides
Th17 cytokines
Inflammation
Host defence
Autoimmune
IL-17B producing cells
Condrocyte, Neuron
IL-17B
IL-17RB
IL-17B targeting cells
Monocyte, Endothelial cell
Inflammatory genes
Proinflammatory activities?
IL-17D producing cells
CD4+ T cell, B cell
IL-17D
?
IL-17D targeting cells
Endothelial cell, Myeloid progenitor
Proinflammatory activities?

B

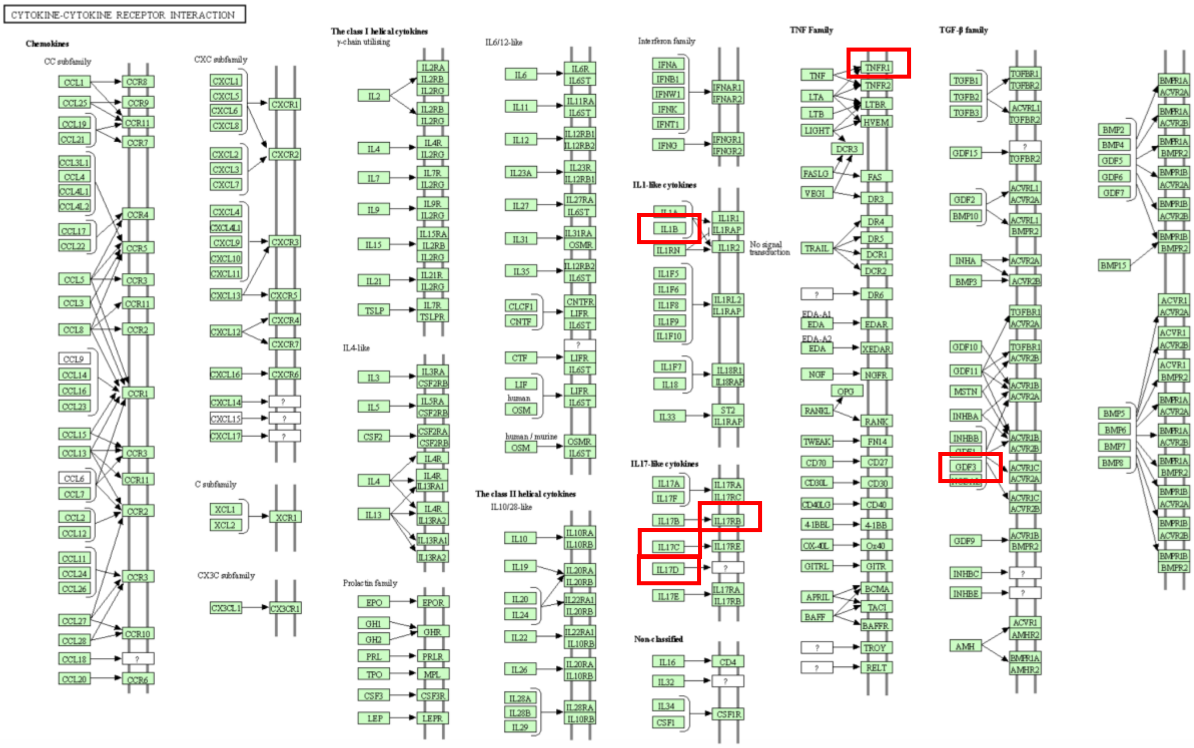
CYTOKINE-CYTOKINE RECEPTOR INTERACTION
Chemokines
CC subfamily
CXC subfamily
C subfamily
CX3C subfamily
The class I helical cytokines
γ-chain utilizing
IL6/12-like
IL4-like
Prolactin family
Interferon family
The class II helical cytokines
IL10/28-like
IL1-like cytokines
No signal transduction
IL17-like cytokines
Non-classified
TNF Family
TGF-β family
IL1B
IL17RB
IL17C
IL17D
TNFR1
GDF3

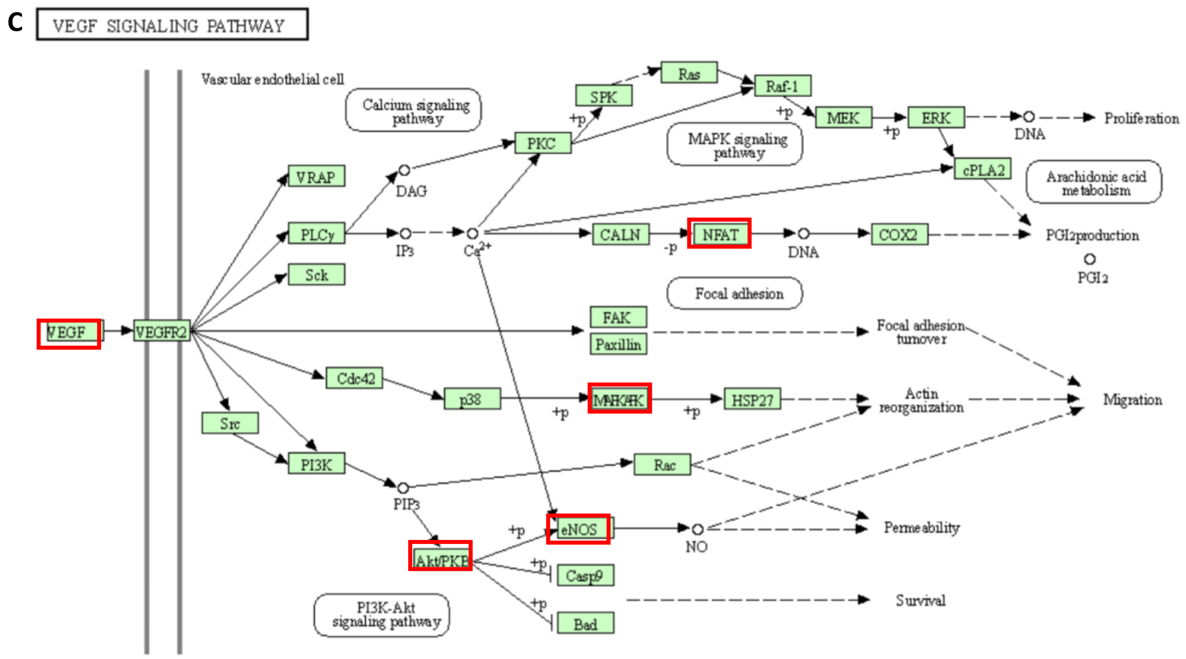

Figure S3. Dementia dysregulated pathways. (A) IL17 signaling pathway. **(B)** Cytokines-cytokines receptors interaction. **(C)** VEGF signaling pathway. In red are the proteins that were differentially expressed in dementia.

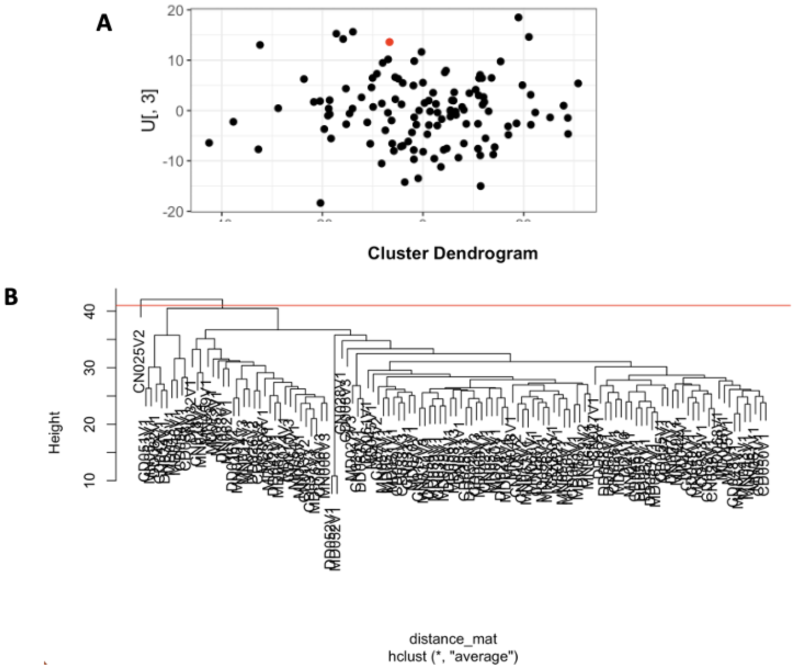

Figure S4. Outliers detection. (A) One sample was identified as an outlier due to a covariate-adjusted PCA value more than four standard deviations from the centroid. **(B)** The same sample was confirmed by clustering.
